# Supplementary figures and images for: Self-supervised neural network improves tri-exponential intravoxel incoherent motion model fitting compared to least-squares fitting in non-alcoholic fatty liver disease
Source: Front Physiol. 2022 Sep 6;13:942495. doi: 10.3389/fphys.2022.942495 (PMC9485997; doi:10.3389/fphys.2022.942495)

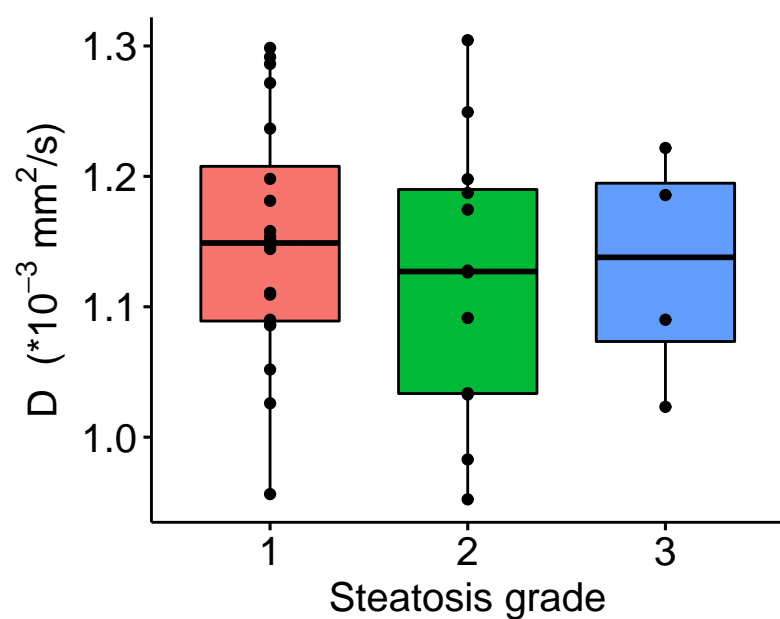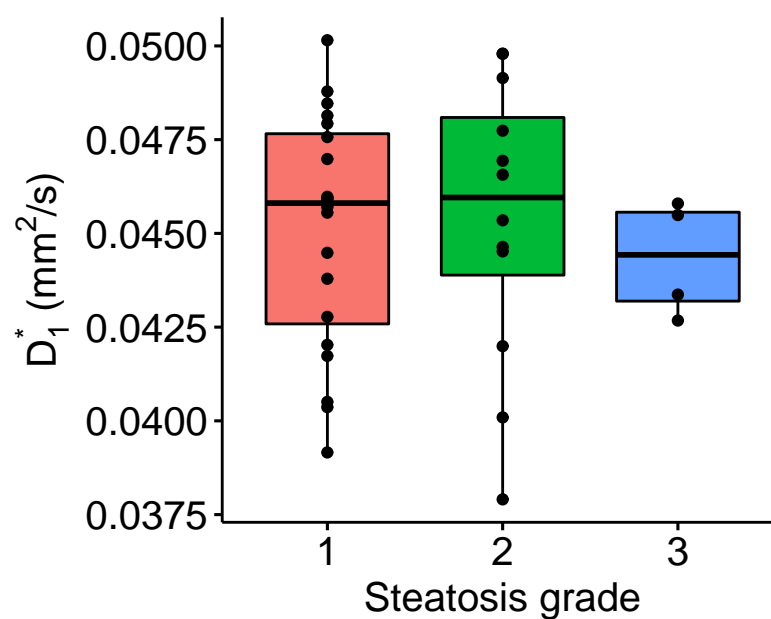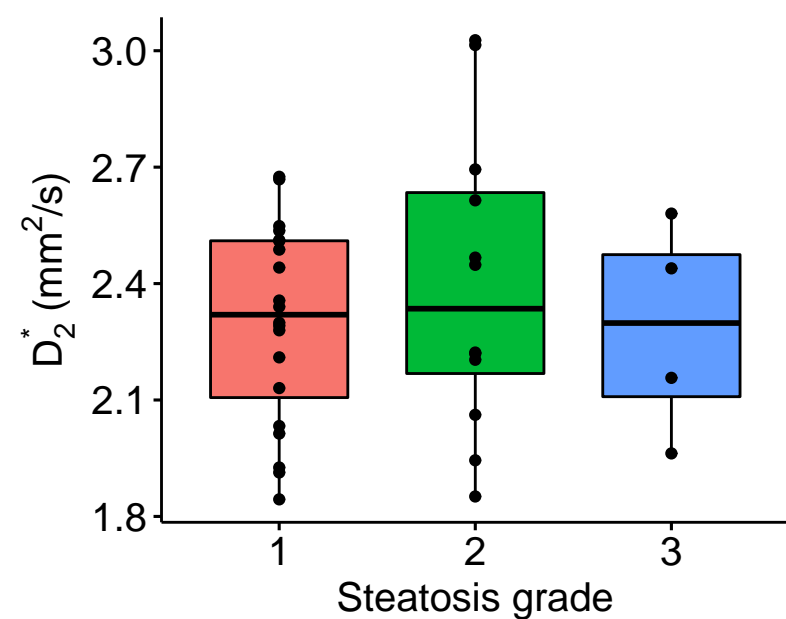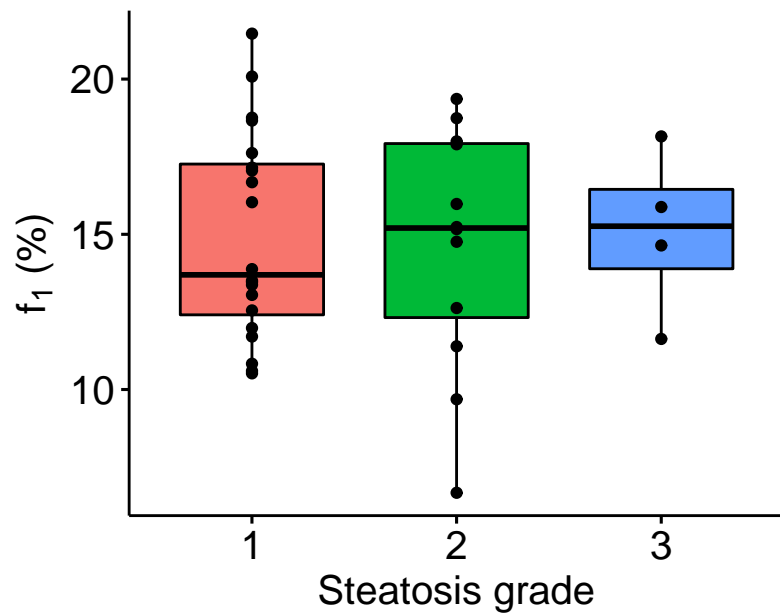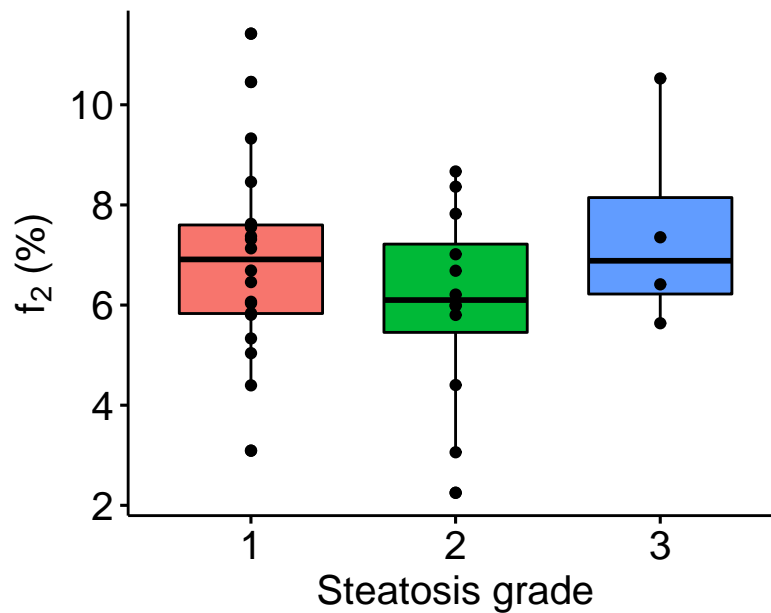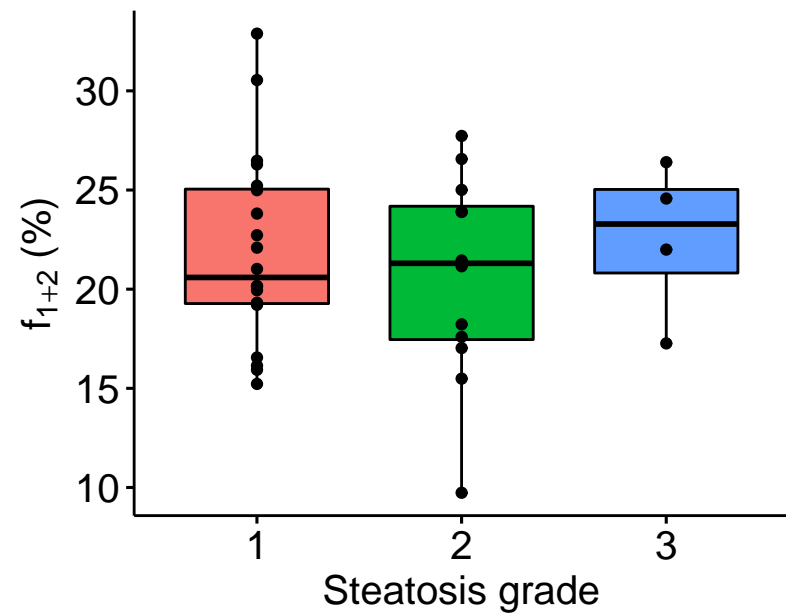

Supplement: Supplementary file 1 [file DataSheet2.PDF]

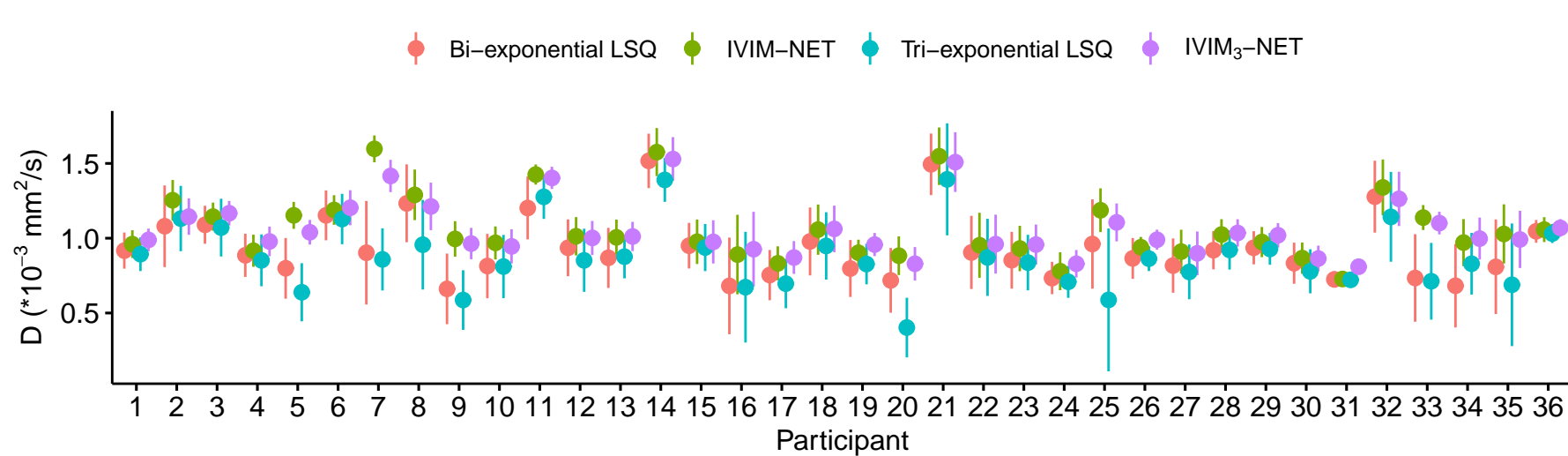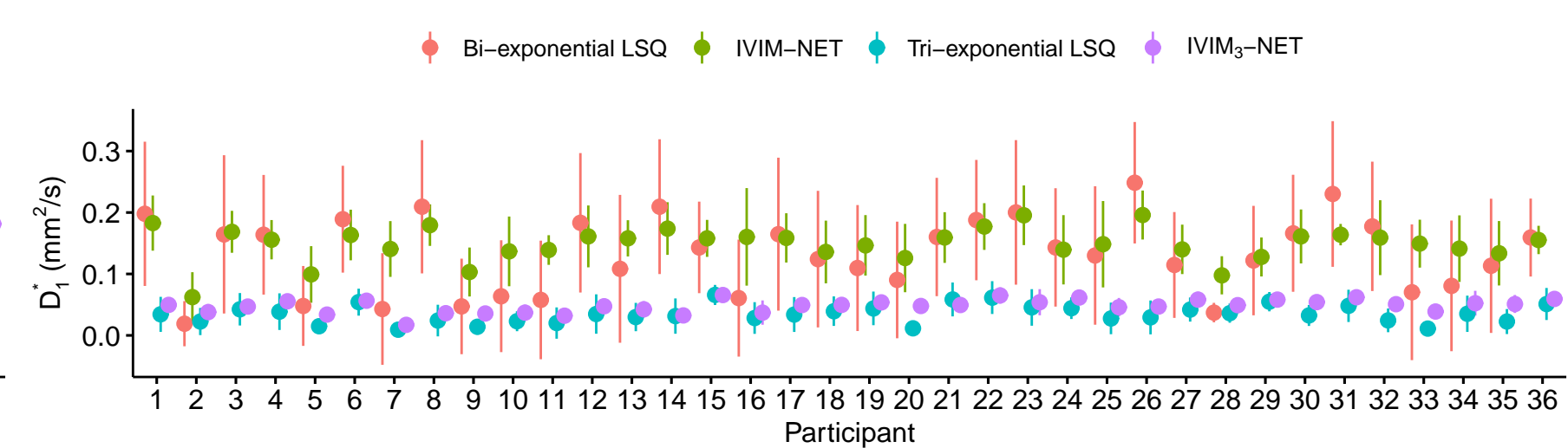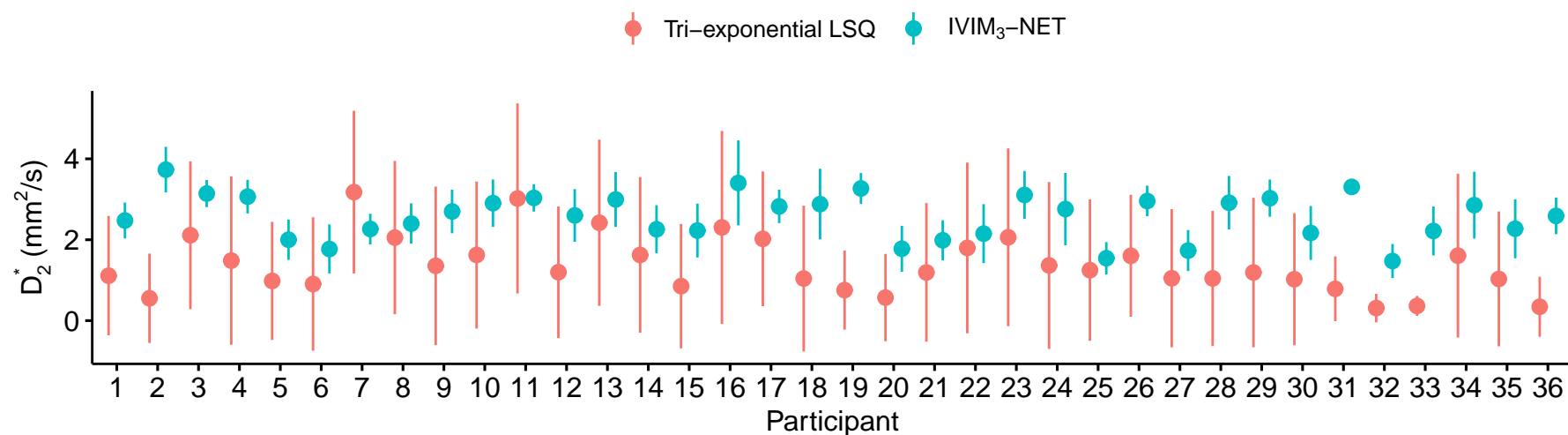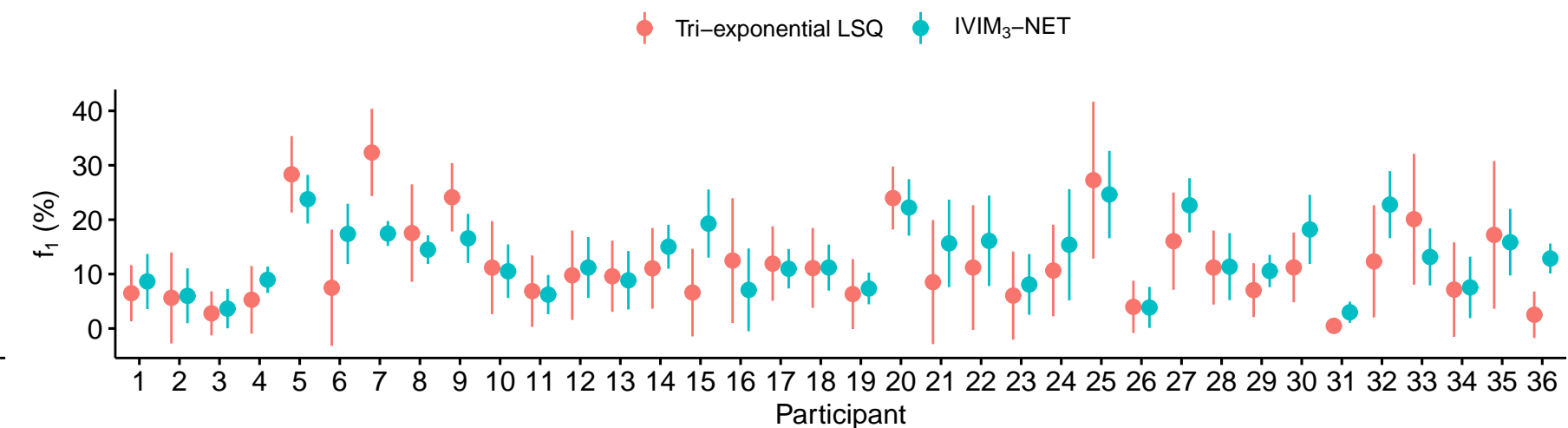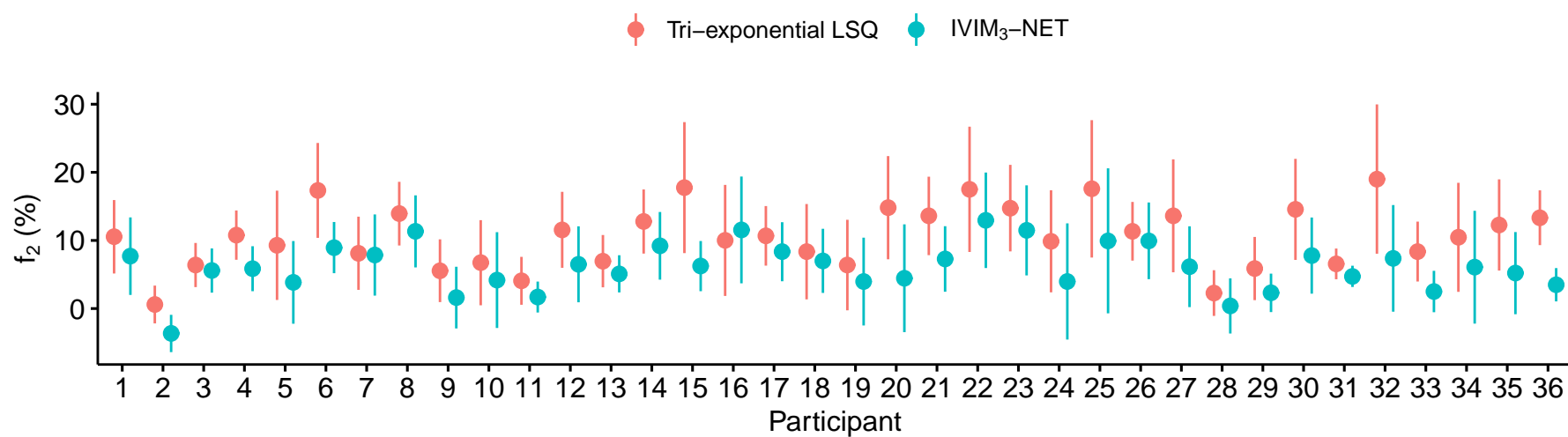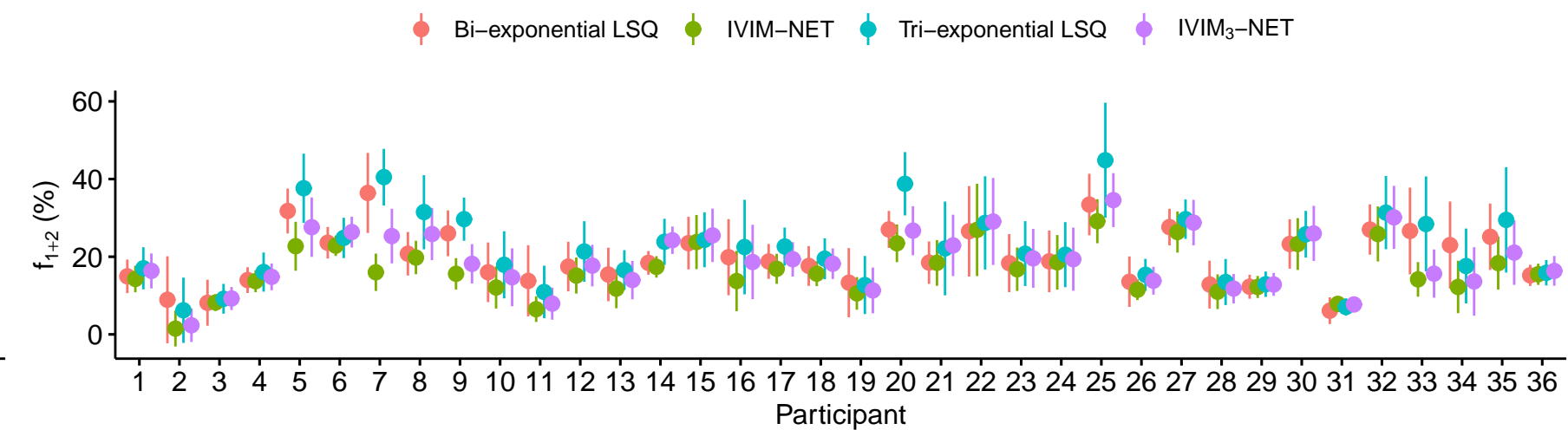

Supplement: Supplementary file 2 [file DataSheet1.PDF]
